# The complete chloroplast genome of *Tetradoxa omeiensis* (Adoxaceae) provides insights into the phylogenetic relationship of Adoxaceae

**DOI:** 10.1080/23802359.2018.1493363

**Published:** 2018-10-12

**Authors:** Yaling Wang, Xiaoyue Yang, Zefu Wang, Hong Chen, Guohua Zhang, Li Chen

**Affiliations:** Life Science and Engineering College, Northwest Minzu University, Lanzhou, China

**Keywords:** *Tetradoxa omeiensis*, chloroplast genome, Endangered species, Hengduan Mountains

## Abstract

The complete chloroplast genome of *Tetradoxa omeiensis*, which is endemic and endangered to the Hengduan Mountains, was sequenced and characterized in this study. The chloroplast genome is 157,502 bp in length, and it is consisted of four distinct regions: a large single-copy region (LSC, 86,526 bp), a small single-copy region (SSC, 18,682 bp) and a pair of inverted repeat regions (IRs, 26,147 bp). A total of 134 genes were annotated, including 86 protein-coding genes (80 PCG species), 40 tRNA genes (33 tRNA species) and 8 ribosomal RNA genes (4 rRNA species). We also reconstructed a comprehensive phylogenetic relationship of the family Adoxaceae, which demonstrated that the three Adoxaceae species clustered into two well-supported clades, and *T. omeiensis* is most closely related to the *Adoxa moschatellina*.

*Tetradoxa omeiensis*, a monotypic plant of the genus *Tetradoxa* (Adoxaceae), is endemic to Hengduan Mountains, where is the southeastern margin of the Qinghai-Tibet Plateau of China (Wu 1981). This species is listed as “Endangered” in the latest IUCN Red List of Threatened Species (http://www.iucnredlist.org/search) and is also an endangered species in China (Wang and Xie 2004). In recent years, the related researches about *T. omeiensis* is rare and conflicting (Mao et al. 2005). The accurate phylogenetic relationship for *T. omeiensis* need to be better studied as a conservation target for further protection and recovery. Herein, we reported the complete chloroplast genome and the gene annotation of *T. omeiensis*. The annotated chloroplast genome has been submitted to GenBank under the accession number of KX258653.

Fresh leaves of a single individual were collected from Erlangshan (Sichuan, China; coordinates: 102°18'9" E, 29°50'41" N). Voucher specimen of the species was deposited in the Key Laboratory of Bio-resource and Eco-environment of Ministry of Education (Sichuan, China). The leaves were used to extract the total genomic DNA with a modified CTAB method (Doyle and Doyle 1987). The whole-genome sequencing was carried out on the Hiseq2500 System (Illumina, USA). In all, about 2 G high-quality base pairs of sequencing data were obtained and used for the genome assembly using Velvet version 1.2.04 (Zerbino and Birney 2008). Then, the resulting contigs were aligned to the reference *Sinadoxa corydalifolia* chloroplast genome (KX258651; Wang et al. 2016) with Samtools (Li et al. 2009) and further processed into a complete genome by Genious version 8.0.5 (Kearse et al. 2012). After filling gaps with GapCloser (Luo et al. 2012), a 157,502-bp chloroplast genome of *T. omeiensis* was obtained. The annotation was performed using Plann (Huang and Cronk 2015). Then, we corrected the annotation with Sequin software (NCBI website). Finally, a physical map was drawn using OGDRAW (http://ogdraw.mpimp-golm.mpg.de/; Lohse et al. 2013; Figure 1).

**Figure 1.**
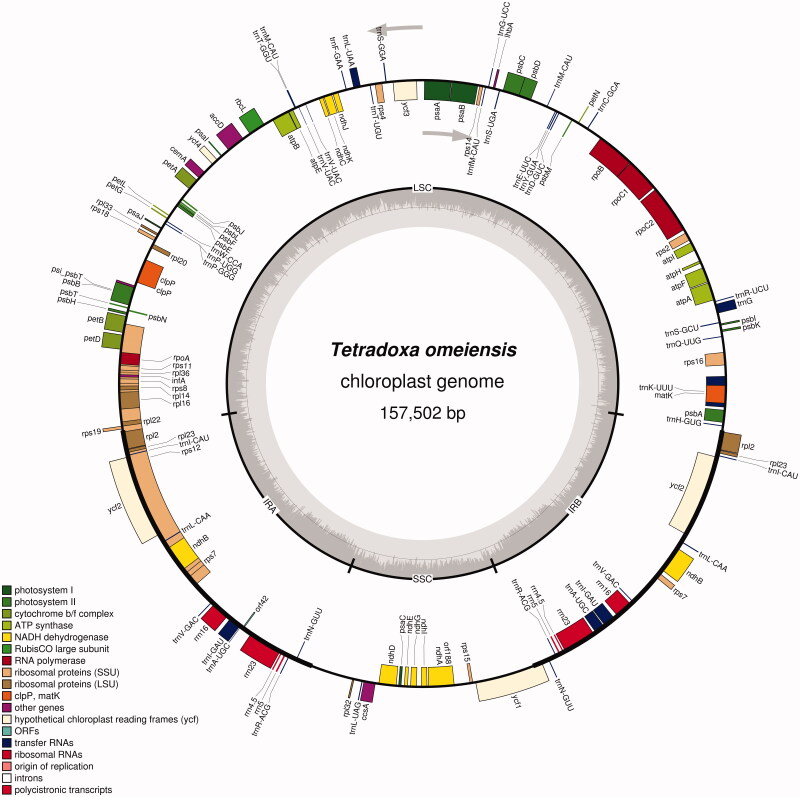
Circular gene map of the chloroplast genome of *T. omeiensis*.

The complete chloroplast genome of *T. omeiensis* has a total length of 157,502 bp with a typical quadripartite structure, consisting of a large single-copy (LSC) region of 86,526 bp, two inverted repeat (IR) regions of 26,147 bp and a small single-copy (SSC) region of 18,682 bp. A total of 134 genes were annotated, including 86 protein-coding genes (80 PCG species), 40 tRNA genes (33 tRNA species) and 8 ribosomal RNA genes (4 rRNA species). The most of gene species occur as a single copy, while 17 gene species occur in double copies, including all rRNA species, 7 tRNA species and 6 PCG species.

We also reconstructed a comprehensive phylogenetic tree of the family Adoxaceae, which consisted of only three genus and each genus contained only one species (Wu 1981), based on their complete chloroplast genome sequences. We aligned the sequences with MAFFT version 7.221 (Katoh and Standley 2013). And the maximum likelihood (ML) tree with 1000 bootstrap replicates was performed with RAxML version 8.1.24 (Stamatakis 2014). The ML phylogenetic tree strongly showed that Adoxaceae species clustered into two well-supported clades, and *T. omeiensis* was closely related to *Adoxa moschatellina* (Figure 2).

**Figure 2.**
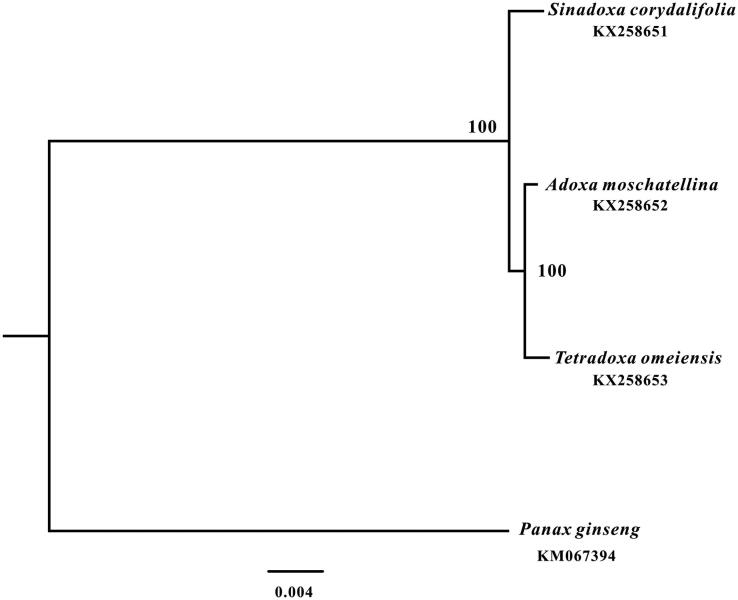
Phylogenetic relationships of Adoxaceae based on the complete chloroplast genomes.

Above all, this report provides essential data for population, phylogenetic, and other studies of *T. omeiensis*, as well as Adoxaceae. A good knowledge of its genomic information can provide insights for development, utilization and evolutionary histories.
